# Free-breathing, non-contrast, three-dimensional whole-heart coronary magnetic resonance imaging for the identification of culprit and vulnerable atherosclerotic plaque

**DOI:** 10.1016/j.jocmr.2025.101898

**Published:** 2025-04-22

**Authors:** Reza Hajhosseiny, Adam Hartley, Graham Cole, Camilla Munoz, Amarjit Sethi, Rasha Al-Lamee, Saud Khawaja, Sameer Zaman, James Howard, Deepa Gopalan, Ben Ariff, Raffi Kaprielian, Radhouene Neji, Karl P. Kunze, Amit Kaura, Claudia Prieto, Ramzi Khamis, René M. Botnar

**Affiliations:** a School of Biomedical Engineering and Imaging Sciences, King’s College London, St. Thomas’ Hospital, London, UK; b National Heart and Lung Institute, Imperial College London, Hammersmith Hospital, London, UK; c MR Research Collaborations, Siemens Healthcare Limited, Camberley, UK; d School of Engineering, Pontificia Universidad Católica de Chile, Santiago, Chile; e Institute for Biological and Medical Engineering, Pontificia Universidad Católica de Chile, Santiago, Chile; f British Heart Foundation Centre of Research Excellence, King's College London, London, UK; g Millennium Institute for Intelligent Healthcare Engineering, Santiago, Chile; h Institute for Advanced Study, Technical University of Munich, Garching, Germany

**Keywords:** Coronary artery disease, Atherosclerosis, Vulnerable plaque, CMR

## Abstract

**Background:**

Detection of vulnerable coronary plaque can predict future myocardial infarctions. We have developed a novel, non-contrast cardiovascular magnetic resonance sequence (iT2prep-BOOST), enabling simultaneous, co-registered coronary angiography and plaque detection.

**Objectives:**

To validate iT2prep-BOOST in patients with non-ST-segment elevation myocardial infarction (NSTEMI).

**Methods:**

41 patients with suspected NSTEMI were recruited. Invasive coronary angiography ± intravascular imaging was used to classify coronary segments into the following categories: normal, non-culprit and culprit segments; stenosed segments as well as segments with vulnerable plaque features (lipid, calcium, fibroatheroma, thin cap fibroatheroma (TCFA), plaque-rupture and thrombus). The plaque/myocardial signal intensity ratio (PMR) in each coronary segment was analyzed on iT2prep-BOOST.

**Results:**

The mean ± standard deviation PMR of culprit segments was significantly higher than non-culprit segments and normal segments (1.01 ± 0.14 vs. 0.67 ± 0.18 vs. 0.35 ± 0.24, P<0.001, respectively). Coronary segments with lipid, calcium, and fibroatheroma had a significantly higher PMR compared to normal coronary segments (P<0.001), but significantly lower than segments with plaque-rupture and intraluminal thrombus (P<0.05). There was a progressive increase in PMR with increasing coronary segment stenosis (P<0.001). There was a significant association on multivariable analysis between HbA1c as well as family history of coronary artery disease and mean PMR (P = 0.05 and P = 0.04, respectively).

**Conclusion:**

iT2prep-BOOST has the potential to simultaneously visualize coronary artery lumen and plaque and differentiate normal segments from non-culprit and culprit plaque segments non-invasively and without contrast. The prognostic value of PMR needs to be investigated in a prospective multicenter study.

## Graphical abstract

**Figure:**
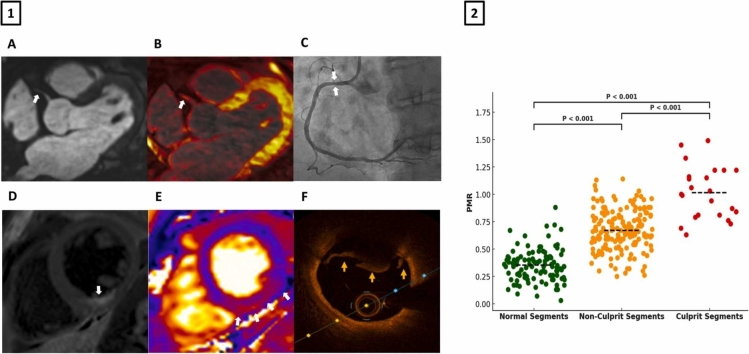
**1.** A patient who presented with chest pain, peak high sensitivity troponin of 13459 ng/L and ST-segment depression in the inferior electrocardiogram leads. **A**-Bright-blood coronary angiography from iT2Prep-BOOST showing a stenosis in the proximal RCA (white arrow). **B**-Co-registered fusion image of iT2Prep-BOOST bright-blood and black-blood datasets showing increased signal from the proximal RCA lesion (white arrow). **C**-Invasive coronary angiography showing a subtle stenosis in the proximal RCA (white arrow). **D**-Dark-blood late gadolinium enhancement short axis image showing a partial thickness infarction affecting the basal inferior wall (white arrows). **E**-Pre-contrast T2 mapping showing increased signal in the basal inferior wall (white arrows). **F**-Optical coherence tomography of the proximal RCA lesion demonstrating acute plaque rupture and intraluminal thrombus (yellow arrows). **2.** Dot strip plot showing the per-segment analysis of the mean ± standard deviation PMR of normal segments, bystander non-culprit segments and culprit segments as identified iT2Prep-BOOST. *PMR* plaque to myocardial signal intensity ratio, *RCA* right coronary artery, *SD* standard deviation.

## 1. Introduction

Coronary artery disease with vulnerable plaque pathology at its center, is the principal cause of cardiovascular mortality worldwide [1]. The early detection and long-term monitoring of vulnerable plaque may enable targeted risk stratification and prophylactic treatment of patients most at risk of an acute coronary syndrome (ACS), whilst accurate culprit plaque identification will increase the precision of coronary intervention [2], [3].

Coronary artery stenosis and recently vulnerable plaque imaging is performed with non-invasive coronary computed tomography angiography (CCTA) or with invasive X-ray coronary angiography and intravascular imaging (intravascular ultrasound (IVUS), optical coherence tomography (OCT) and near infra-red spectroscopy) [4], [5], [6], [7]. However, beam-hardening and calcium-blooming artefacts, the inability to follow breath-hold instructions and risk of iodinated contrast mediated nephropathy pose significant challenges [8]. Furthermore, long-term cumulative risk from ionizing radiation as well as invasive complications limit the interval and absolute number of follow up monitoring scans.

Alternatively, coronary plaque and thrombus can be identified on non-contrast T1-weighted (T1W) cardiovascular magnetic resonance (CMR) as high intensity plaque, possibly through T1 shortening properties of plaque components (intraplaque haemorrhage, thrombus, lipid, macrophages and fibroatheroma) and is significantly associated with future coronary events, independent of coronary lumen stenosis [9], [10], [11]. It could potentially be a biomarker to predict risk, guide interventional treatment and monitor the treatment effects of therapeutic agents such as statins [12].

Current implementations of CMR high intensity plaque imaging either provide a heavily T1 weighted black blood 3D volume optimized for coronary thrombus or intraplaque haemorrhage detection but with poor anatomy visualization (including the coronary vessel wall) [9] or simultaneous acquisition of a heavily T1 weighted black blood 3D volume with an anatomical reference volume for coronary lumen visualization, using the so-called CATCH sequence [11], [10]. While providing a co-registered black blood and anatomical reference volume and high contrast visualization of intraplaque hemorrhage and thrombus, the CATCH sequence suffers from low coronary wall to blood contrast and low contrast between the coronary lumen and adjacent myocardium. Furthermore, respiratory motion correction only considers affine inter-bin motion thereby neglecting beat-to-beat intra-bin and 3D non-rigid inter-bin motion which may lead to remaining motion artefacts in patients with complex breathing patterns.

To address these shortcomings, we have recently developed a novel image-navigated (iNAV), three-dimensional (3D), free-breathing, non-contrast CMR sequence (iT2prep-BOOST) that performs a beat-to-beat translational intra-bin motion correction followed by a 3D non-rigid motion corrected iterative SENSE reconstruction to minimise both intra and inter-bin motion. Moreover, we employ a T2-prepared inversion recovery(T2prep-IR)—no prep pre-pulse combination that allows for simultaneous high-resolution and high-contrast visualization of the coronary lumen and plaque on two fully co-registered bright-blood (angiography) and black-blood (plaque-assessment) datasets, with 100% respiratory scan efficiency, advanced 3D motion correction, and a highly accelerated and predictable acquisition time of ≈ 10 min [13], [14], [15], [16], [17]. Finally, we propose a patch based low-rank denoising framework in combination with a non-rigid motion corrected iterative sense reconstruction to further enhance image quality and to enable higher undersampling factors.

In this clinical study, we investigate the potential of iT2prep-BOOST for plaque detection and characterization in patients with suspected non-ST-segment elevation myocardial infarction (NSTEMI).

## 2. Methods

### 2.1. Study design

This was a prospective single center study of patients with suspected NSTEMI who were approached to undergo an iT2prep-BOOST scan prior to invasive coronary angiography ± intravascular imaging at Imperial College Healthcare NHS Trust, London, United Kingdom. Written informed consent was obtained from each patient prior to enrolment. The study was approved by the local institutional review board and the United Kingdom National Research Ethics Committee (reference number: 246728).

### 2.2. Study population

Patients with suspected NSTEMI (detection of a rise and/or fall of cardiac troponin values with at least one value above the 99th percentile upper reference limit and with at least one of the following: new symptoms of acute myocardial ischaemia (chest pain and/or shortness of breath); new ischaemic electrocardiogram (ECG) changes without ST segment elevation or new left bundle branch block (LBBB); development of pathological Q waves) [18] were recruited between the May 1, 2021 and June 1, 2022.

Exclusion criteria included patients with pacemaker, cochlear implants, cerebral aneurysm clip, implanted electronic device, claustrophobia, severe left ventricular dysfunction (left ventricular ejection fraction [LVEF]<35%), cardiogenic shock, atrial fibrillation, pregnancy, significant renal impairment (estimated glomerular filtration rate [eGFR]<30 mL/min/1.73 m^2^) or previous percutaneous coronary intervention (PCI).

### 2.3. Study protocol

#### 2.3.1. iT2prep-BOOST coronary artery imaging

All iT2prep-BOOST CMR acquisitions were performed on a 1.5T MRI scanner (MAGNETOM Aera, Siemens Healthineers, Erlangen, Germany) with a dedicated 32-channel spine coil and an 18-channel body coil. In the absence of contraindications, each patient was treated with intravenous metoprolol (Betaloc ®, AstraZeneca, Cambridge, United Kingdom), in 5 mg increments via a peripheral intravenous cannula with a maximum dose of 30 mg, aiming for a target heart rate <65 bpm in order to maximize the diastolic acquisition window, reduce heart rate variability and cardiac motion artefacts. All patients were given 800 micrograms of sublingual glyceryl trinitrate to promote coronary vasodilation unless the systolic blood pressure was less than 100 mmHg.

A 3-lead vector ECG was used for cardiac synchronization. To determine the mid-diastolic or end-systolic rest period, a free-breathing axial cine was acquired in a four-chamber orientation. Patient-specific trigger delay and acquisition window (<100 ms), set to correspond with minimized motion of the visualized right coronary artery and mitigate the effect of cardiac motion (mid-diastole if the heart rate was <65bpm or end-systole if the heart rate was ≥65bpm).

**Fig. 1:**
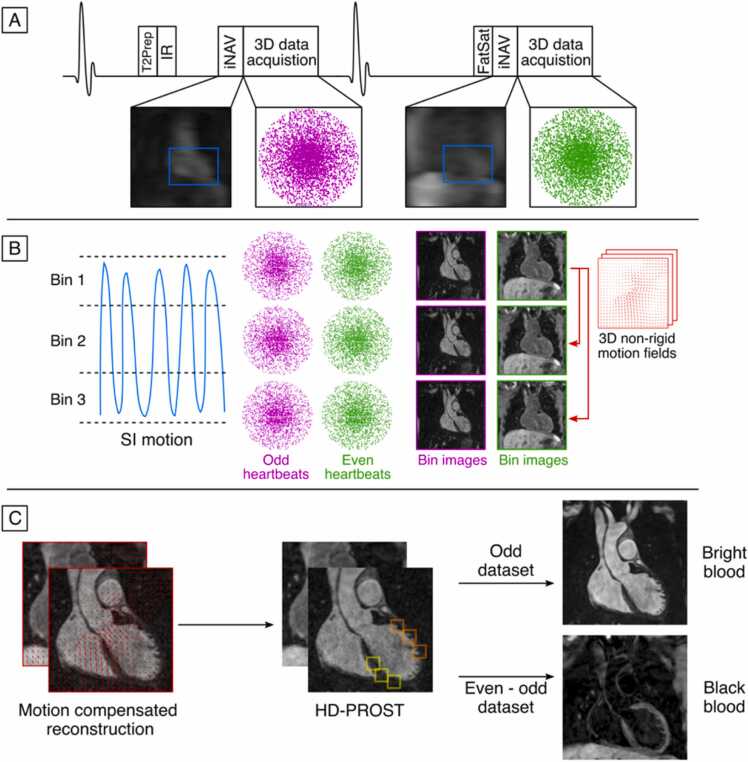
Proposed iT2Prep-BOOST acquisition and reconstruction scheme. **A-**3D data acquisition is performed in an interleaved fashion, with a combined T2Prep-IR preparation pulse used in odd heartbeats; and only fat saturation used in even heartbeats. The sequence iNAVs for motion compensation and 100% respiratory scan efficiency. **B-**SI motion estimated from the iNAVs is used to bin the data and produce respiratory-resolved 3D bin images, which are then used to estimate 3D non-rigid motion. **C-**Co-registered motion-corrected 3D images are obtained by incorporating non-rigid motion and low-rank patch-based denoising into the reconstruction, which are finally used to obtain the bright-blood data for lumen visualization (odd heartbeats) and black-blood data for vessel wall visualization (by direct subtraction of even and odd heartbeats). *3D* three dimensional, *SI* superior inferior, *T2Prep-IR* T2-preparation and inversion recovery, *iNAVs* image navigators

Data acquisition with iT2Prep-BOOST consists of an ECG-triggered interleaved 3D balanced steady-state-free-precession (SSFP) research sequence (Fig. 1), where a combined T2-preparation and inversion recovery (T2Prep-IR) [19] preparation module is applied before data acquisition in odd heartbeats (T2Prep duration of 40 ms and TI = 110ms) [20] and only fat saturation is applied in even heartbeats, resulting in two bright-blood datasets [14]. The proposed iT2Prep-BOOST scan was acquired with the following parameters: bSSFP readout, coronal orientation, field-of-view [FOV] 320 × 320 × 80–120 mm, spatial resolution 1.2 mm isotropic, 3–4 fold undersampling factor, flip angle 90º, TR/TE = 2.94/1.47 ms, bandwidth = 965 Hz/pixel.

The image reconstruction framework consists of three steps as follows: (1) beat-to-beat respiratory binning and intra-bin 2D translational motion correction using iNAV to enable 100% respiratory scan efficiency (without data rejection) and predictable scan time [15], [21]; (2) bin-to-bin 3D non-rigid motion estimation and subsequent non-rigid motion corrected iterative SENSE reconstruction [16]; and (3) to minimize artefacts arising from the undersampled acquisition, a low-rank patch-based denoising (HD-PROST) was used [22], [23]. Steps 1 and 2 were performed in-line on the scanner, while step 3 was performed offline.

The first bright-blood dataset can be used for coronary lumen visualization, while direct subtraction of the two bright-blood datasets is used to create the co-registered black-blood dataset for plaque characterization (Fig. 1).

#### 2.3.2. Invasive X-ray coronary angiography and intravascular imaging

Immediately after completing their CMR examination, patients were transferred to the coronary catheterization laboratory to undergo their scheduled invasive coronary angiography as part of their routine clinical treatment plan. The decision to perform intravascular imaging was applied on a lesion by lesion basis, at the clinical discretion of the treating interventional cardiologist (who was blinded to the iT2Prep-BOOST analysis), based on patient-specific factors, decision to proceed with PCI and technical factors such as overall disease burden, degree of stenosis, calcification, vessel tortuosity, and lumen diameter. The OCT catheter system used was a Dragonfly™ Optis Imaging Catheter (Abbott Medical, Abbott Park, Illinois). The IVUS catheter system used was the Eagle Eye Platinum IVUS Catheter (Volcano Corporation, San Diego, California).

### 2.4. Coronary data analysis

All datasets were analyzed using a nine segment coronary model, described in previous studies [9], [24], [25], [26]. This divides the coronary arterial tree into the following segments: left main stem (LMS), left anterior descending (LAD) artery (proximal, middle and distal), right coronary artery (RCA) (proximal, middle and distal), and left circumflex (LCx) artery (proximal and distal).

#### 2.4.1 Invasive coronary artery analysis

All diagnostic coronary segments on invasive coronary angiography were assessed on Medcon (Medcon Ltd., Tel Aviv, Israel) by four interventional cardiologists with more than 5 years of experience who were blinded to the CMR data. Individual coronary segments were assigned to the following categories: normal segments (segments with no stenosis or plaque features identified on invasive coronary angiography and or intravascular imaging), segments with bystander non-culprit plaque (segments with a degree of stenosis and or plaque features on invasive coronary angiography and or intravascular imaging not meeting the definition of culprit segments), segments with culprit plaque (segments with plaque rupture and or intraluminal thrombus on intravascular imaging and or coronary angiography [26], [27]), segment with 1–24% stenosis, segment with 25–49% stenosis, segment with 50–69% stenosis, segment with >70% stenosis, segment with intraluminal thrombus and segment with calcified plaque. Segments with intravascular imaging were further qualitatively assigned into the following categories: segment with thin cap fibroatheroma (TCFA), segment with lipid rich plaque, segment with acute plaque rupture and segment with fibroatheroma.

#### 2.4.2. CMR coronary artery analysis

All diagnostic coronary segments on iT2Prep-BOOST were analysed on Osirix software (Osirix, Pixmeo SARL, Bernex, Switzerland) by two imaging cardiologists with level III accreditation in CMR with more than 3 years of experience, who were blinded to the invasive coronary imaging analysis, with the average score of the two readers used for analysis. The method to evaluate CMR coronary plaque images has been described previously [9], [11], [28]. The fusion feature of the Osirix workstation was used by overlaying the co-registered black-blood images onto the reference bright blood CMRA images (even heartbeat) to enable simultaneous plaque visualization and coronary artery localization. Plaque-myocardial signal ratio (PMR), was calculated as the signal intensity of the coronary plaque/vessel wall divided by that of nearby left ventricular myocardium, measured using a free-hand region of interest on the co-registered black-blood images. The mean signal intensity detected in each plaque/vessel wall was considered the PMR value for that plaque/vessel wall in a segment-based analysis (supplementary Fig. 3). In a patient-based analysis, the segment with the highest mean PMR value was considered to be the maximum PMR for that patient, whilst the mean PMR for that patient was considered to be the sum of the individual mean PMR for each segment divided by the total number of segments measured for that patient.

### 2.5. Statistical analysis

Continuous variables are presented as mean ± standard deviation or as median and interquartile range depending on the distribution. Categorical variables are expressed as frequency (%). Comparison of more than two independent variables were analysed by analysis of variance (ANOVA). Intraclass correlation coefficients with 95% confidence intervals (CIs) were calculated to assess intra-reader and inter-reader agreement for PMR, with a value >0.80 indicating good agreement.

Restricted cubic spline ordinary least squares regression analysis or linear regression modelling was used to evaluate the independent contribution of patient demographics (age and gender) or cardiovascular risk factors (total cholesterol, low-density lipoprotein (LDL) cholesterol, triglyceride, hemoglobin A1c (HbA1c), hypertension, smoker and family history of coronary artery disease [CAD]) with mean PMR. Similar models were constructed with maximum PMR as the dependent variable. Three knots were used for all restricted cubic spline analyses. For linear regression analysis, each variable was first entered into a univariable model, and those found to be significant at a level of P<0.20 were subsequently entered into a multivariable model. In the multivariable regression analysis, we computed the variance inflation factor (VIF) to address multicollinearity, especially among the lipid profile variables. All the VIF scores were found to be less than 2, indicating that multicollinearity was not a significant concern. All statistical analysis was performed using IBM SPSS Statistics, version 28.0.1.1 (IBM, Armonk, New York) or R statistical package, 3.5.3 (R Foundation for Statistical Computation, Vienna, Austria).

## 3. Results

In total, 44 patients were enrolled into the study. Three patients were excluded (two patients self-discharged from the hospital prior to the invasive coronary angiogram and one patient could not tolerate a CMR scan). All iT2Prep-BOOST acquisitions were successfully completed within an average scan time of 10.8 ± 1.3 mins (range 7.8–14.1 mins), with 100% respiratory scan efficiency. Overall, 25/41 (61%) of patients were scanned with mid-diastolic imaging and 16/41 (39%) of patients were scanned with end-systolic imaging.

**Fig. 2:**
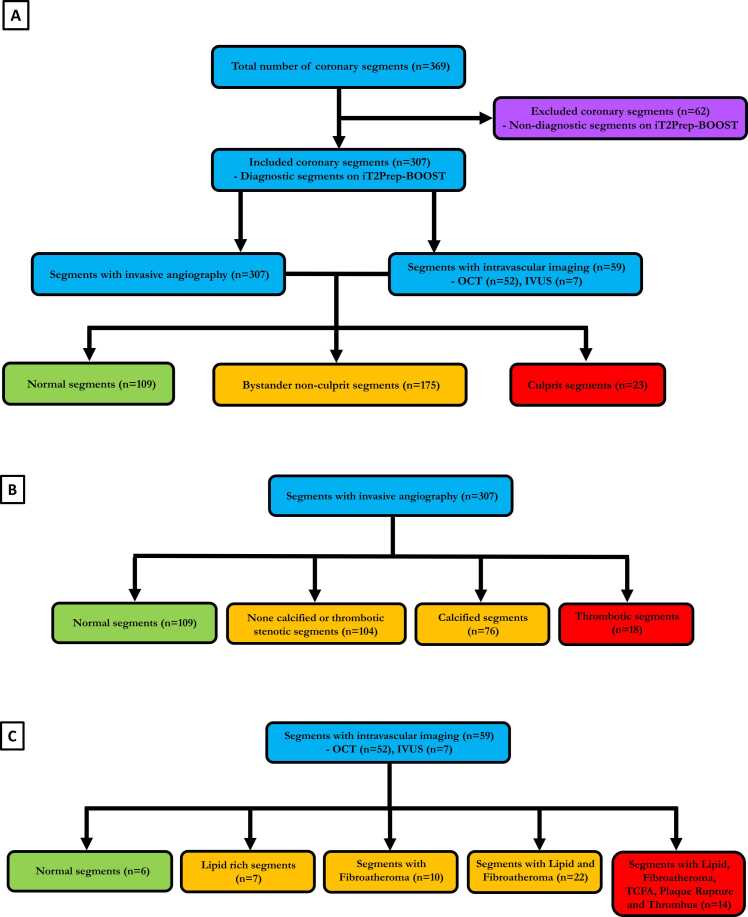
**A:** Flow-chart showing the coronary segment classification with iT2Prep-BOOST, invasive coronary angiogram and intravascular imaging. There were a total of 307 segments that were analyzed in this study. 109 coronary segments were classified as normal (i.e. no stenosis or plaque features identified on invasive coronary angiography and or intravascular imaging). 175 segments were classified as bystander non-culprit segments (i.e. segments with a degree of stenosis and or plaque features not meeting the definition of culprit segments). 23 Segments were classified as culprit segments (plaque rupture or intraluminal thrombus on intravascular imaging and/or invasive coronary angiography). Segments that were exclusively classified with invasive angiography alone (e.g. normal segments, none calcified or thrombotic stenotic segments, calcified segments and thrombotic segments) were assigned accordingly (**B**). Furthermore, 59 segments were classified with intravascular imaging, benefitting from additional plaque classification (e.g. TCFA, segment with lipid rich plaque, segment with acute plaque rupture and segments with fibroatheroma) (**C**). This classification system ensured that every coronary segment could be classified based on the minimum invasive angiography identifiable features, with at least 59 segments benefiting from additional intravascular imaging classification, avoiding selection bias. *OCT* optical coherence tomography, *IVUS* intravascular ultrasound, *TCFA* thin cap fibroatheroma

A total of 19/41 (46%) of patients had intravascular imaging, involving 24 separate vessels (21 with OCT and 3 with IVUS) and 59 individual segments (52 with OCT and 7 with IVUS). Overall, 8/120 (5%), 12/82 (15%) and 42/123 (34%) of proximal, middle and distal coronary segments respectively were deemed non-diagnostic on black-blood iT2Prep-BOOST, leaving a total of 307 coronary segments to be analysed for PMR (Fig. 2). The intrareader intraclass correlation coefficient of PMR was 0.96 (95% CI: 0.93 to 0.98). The interreader intraclass correlation coefficient of PMR was 0.92 (95% CI: 0.80 to 0.97), indicating excellent intra-observer and inter-observer agreement.

Table 1, Table 2 summarize the baseline patient and biochemical characteristics.

**Table 1:** Baseline patient characteristics.

Characteristics	All Patients (n=41)
Male n (%)	31 (76)
Mean age (years)±SD	58.1±14.1
Mean Height (cm)±SD	168.9±9.5
Mean Weight (kg)±SD	79.2±16.3
Mean BMI (kg/m^2^)±SD	27.9±6.2
Diabetes n (%)	15 (37)
Hypertension n (%)	17 (42)
Smoker n (%)	18 (44)
Hyperlipidemia n (%)	29 (71)
Family History of CAD n (%)	17 (42)
History of PVD	0 (0)
History of CVD	2 (5)
Statin use n (%)	14 (34)
Presenting complaint	
Chest Pain n (%) Shortness of Breath n (%)	39 (95) 9 (22)
Significant Disease (>50% stenosis) n (%) 1 vessel disease n (%) 2 vessel disease n (%) 3 vessel disease n (%)	27 (66) 11 (27) 5 (12) 11 (27)

Data are expressed as mean ± standard deviation (SD) or n (%). *BMI* body mass index, *CAD* coronary artery disease, *CVD* cardiovascular disease, *PVD* peripheral vascular disease

**Table 2:** Baseline biochemistry data expressed as mean ± standard deviation.

Biochemistry	Mean±SD
Admission Troponin (ng/L)	904.6±2192.1
Peak Troponin (ng/L)	4329.6±9065.5
Hb (g/L)	142.8±18.3
CRP (mg/L)	5.7±10.4
Creatinine (umol/L)	81.8±23.3
eGFR (ml/min/1.73m^2^)	80.8±14.9
Total Cholesterol (mmol/L)	5.2±1.3
LDL Cholesterol (mmol/L)	3.2±1.2
HDL Cholesterol (mmol/L)	1.2±0.4
Triglycerides (mmol/L)	1.8±1.0
HBA1c	46.5±13.2

*Hb* hemoglobin, *CRP* C-reactive protein; *eGFR* estimated glomerular filtration rate, *LDL* low density lipoprotein, *HDL* high density lipoprotein

Data expressed as mean ± standard deviation (SD)

### 3.1. iT2Prep-BOOST culprit plaque identification

**Fig. 3:**
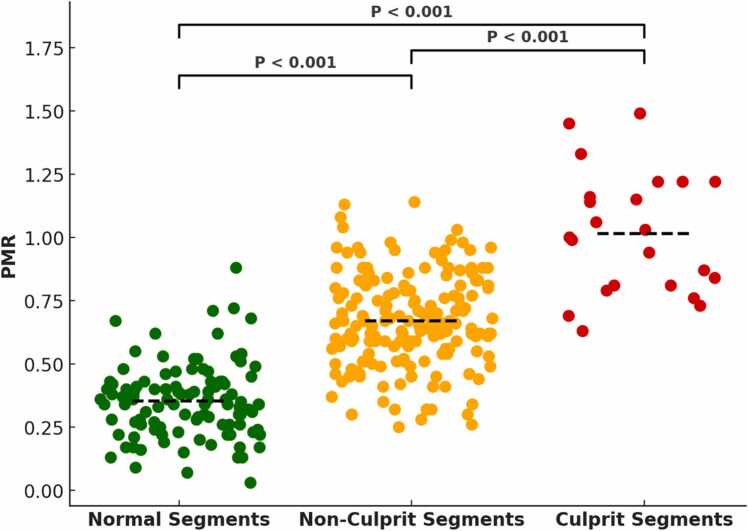
Dot strip plot showing the per segment analysis of the mean ± standard deviation PMR of normal segments (0.35 ± 0.14), bystander non-culprit segments (0.67 ± 0.18) and culprit segments (1.01 ± 0.24) as identified on iT2Prep-BOOST and classified by invasive coronary angiography and intravascular imaging. *PMR* plaque to myocardial signal intensity ratio

iT2Prep-BOOST demonstrated the potential to classify coronary segments into normal, non-culprit and culprit segments based on the mean PMR (Fig. 3). In total, there were 109/307 (36%) normal segments, 175/307 (57%) bystander non-culprit segments and 23/307 (7%) culprit segments as identified by invasive coronary angiography and/or intravascular imaging. The mean PMR of culprit segments was significantly higher than non-culprit segments and normal segments (P<0.001, respectively).

### 3.2. iT2Prep-BOOST plaque characterization

#### 3.2.1. Invasive coronary angiography

**Fig. 4:**
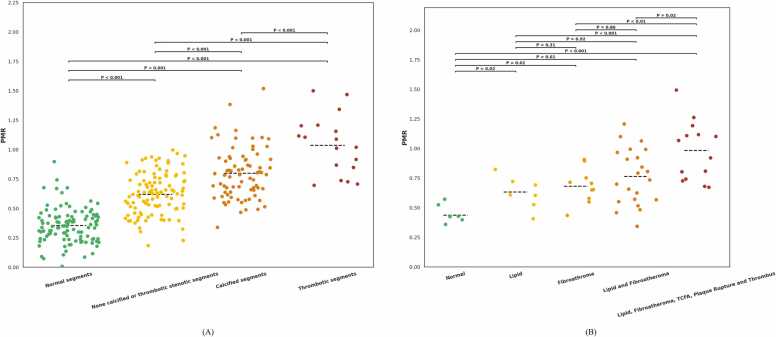
**A:** Dot strip plot showing the per segment analysis of the mean ± SD PMR of normal segments (0.35 ± 0.14), none calcified or thrombotic stenotic segments (0.62 ± 0.17), segments with calcified plaque (0.80 ± 0.21) and segments with intraluminal thrombus (1.03 ± 0.24) as identified on iT2Prep-BOOST and classified by invasive coronary angiography. **B:** Dot strip plot showing the per segment analysis of the mean ± SD PMR of normal segments (0.44 ± 0.11), segments with lipid rich plaque only (0.63 ± 0.14); segments with fibroatheroma only (0.68 ± 0.13); segments with both lipid rich plaque and fibroatheroma (0.76 ± 0.24); and segments with all of lipid rich plaque, fibroatheroma TCFA, acute plaque rupture and intraluminal thrombus (0.98 ± 0.24) as identified on iT2Prep-BOOST and classified by intravascular imaging. *PMR* plaque to myocardial signal intensity ratio, *TCFA* thin cap fibroatheroma, *SD* standard deviation, *PMR* plaque to myocardial signal intensity ratio

iT2Prep-BOOST could characterize coronary segments according to their plaque composition and mean PMR (Fig. 4). The mean PMR of normal segments (n=109);, none calcified or thrombotic stenotic segments (n=104); segments with calcified plaque (n=76) and segments with intraluminal thrombus (n=18) as identified by invasive coronary angiography are shown in Fig. 4A. The mean PMR of none calcified or thrombotic stenotic segments, segments with calcified plaque and segments with intraluminal thrombus were all significantly higher than that of normal segments (P<0.001 respectively). The mean PMR of segments with calcified plaque was significantly higher than that of segments with none calcified or thrombotic stenotic segments (P<0.001). The mean PMR of segments with intraluminal thrombus was significantly higher than that of none calcified or thrombotic stenotic segments as well as segments with calcified plaque (P<0.001, respectively).

#### 3.2.2. Intravascular imaging

The mean PMR of normal segments (n=6); segments with lipid rich plaque only (n=7); segments with fibroatheroma only (n=10); segments with a combination both lipid rich plaque and fibroatheroma (n=22); segments with a combination of all of lipid rich plaque, fibroatheroma, TCFA, acute plaque rupture and intraluminal thrombus (n=14) as identified by intravascular imaging are shown in Fig. 4B. There were no significant differences in the mean PMR of segments with lipid rich plaque only, segments with fibroatheroma only and segments with a combination both lipid rich plaque and fibroatheroma (P = 0.31, P = 0.86 and P = 0.92, respectively). However, the mean PMR of segments with a combination of all of lipid rich plaque, fibroatheroma, TCFA, acute plaque rupture and intraluminal thrombus was significantly higher than that of segments with lipid rich plaque only (P<0.001), segments with fibroatheroma only (P = 0.002) and segments with a combination both lipid rich plaque and fibroatheroma (P = 0.02).

#### 3.2.3. Quantifying plaque burden with iT2Prep-BOOST

**Fig. 5:**
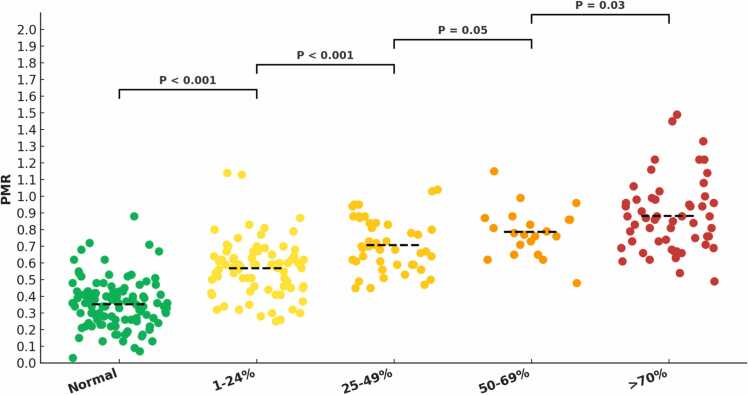
Dot strip plot showing the per segment analysis of the mean ± standard deviation PMR of normal segments (0.35 ± 0.14), segments with 1–24% stenosis (0.57 ± 0.17), segments with 25–49% stenosis (0.71 ± 0.16), segments with 50–69% stenosis (0.79 ± 0.15), and segments with >70% stenosis (0.88 ± 0.22) as identified on iT2Prep-BOOST and classified by invasive coronary angiography. *PMR* plaque to myocardial signal intensity ratio

iT2Prep-BOOST could assess atherosclerotic disease burden according to the mean PMR (Fig. 5). The PMR of normal segments [109/307 (36%)], segments with 1–24% stenosis [78/307 (25%)], segments with 25–49% stenosis [44/307 (14%)], segments with 50–69% stenosis [21/307 (7%)], and segments with >70% stenosis [55/307 (18%)] as identified by invasive coronary angiography are shown in Fig. 5. The mean PMR significantly increased with increasing degree of segment stenosis.

#### 3.2.4. Association of PMR with cardiovascular risk factors

Restricted cubic spline analyses showed a near linear relationship between HbA1c, total cholesterol, LDL cholesterol and triglyceride level with either mean PMR (Supplementary Fig. 1) or maximum PMR (Supplementary Fig. 2). Whilst there was an association between cardiovascular risk factors (HbA1c, total cholesterol, hypertension, and family history of CAD) and either mean or maximum PMR on univariable analysis (Table 3, Table 4), the only significant associations on multivariable analysis were between HbA1c and mean PMR (P = 0.05) and between family history of CAD and mean PMR (P = 0.04), (Table 3 and Supplementary Fig. 3).

**Table 3:** Univariable and multivariable linear regression analysis of the association between cardiovascular risk factors and mean PMR.

Univariable analysis for mean PMR outcome				Multivariate analysis for mean PMR outcome			
Variable	B-coefficient	95% CI	P-value	Variable	B-coefficient	95% CI	P-value
Age	0.0048	0.00055 to 0.0091	0.03	Age	0.0034	0.00093 to 0.0078	0.12
Gender	0.087	−0.057 to 0.23	0.20	Gender	0.031	−0.096 to 0.16	0.62
Total cholesterol	0.026	−0.024 to 0.075	0.30				
LDL cholesterol	0.021	−0.033 to 0.074	0.44				
Triglyceride	0.053	−0.0077 to 0.11	0.09	Triglyceride	0.040	−0.017 to 0.098	0.16
HbA1c	0.0079	0.0038 to 0.012	<0.001	HbA1c	0.0047	0.00013 to 0.0093	0.05
Hypertension	0.15	0.035 to 0.27	0.01	Hypertension	0.035	−0.094 to 0.16	0.58
Smoker	0.044	−0.082 to 0.17	0.49				
FHx of CAD	0.15	0.035 to 0.27	0.01	FHx of CAD	0.11	0.0070 to 0.22	0.04

Each variable was first entered into a univariable model, and those found to be significant at a level of P<0.20 were subsequently entered into a multivariable model. *PMR* plaque to myocardial signal intensity ratio, *LDL* low density lipoprotein, *HDL* high density lipoprotein, *FHx* family, *CAD* coronary artery disease

Data are expressed as B-coefficient with 95% confidence intervals (CI) with P values.

**Table 4:** Univariable and multivariable linear regression analysis of the association between cardiovascular risk factors and maximum PMR.

Univariable analysis for maximum PMR outcome				Multivariate analysis for maximum PMR outcome			
Variable	B-coefficient	95% CI	P-value	Variable	B-coefficient	95% CI	P-value
Age	0.00092	−0.0051 to 0.0069	0.76				
Gender	0.076	−0.12 to 0.27	0.43				
Total cholesterol	0.072	0.010 to 0.13	0.023	Total cholesterol	0.0096	−0.14 to 0.33	0.50
LDL cholesterol	0.067	−0.00081 to 0.14	0.053	LDL cholesterol	0.038	−0.28 to 0.20	0.76
Triglyceride	0.078	−0.0015 to 0.16	0.054	Triglyceride	0.025	−0.079 to 0.13	0.63
HbA1c	0.0056	−0.00061 to 0.012	0.076	HbA1c	0.0048	−0.0016 to 0.011	0.13
Hypertension	0.016	−0.15 to 0.18	0.85				
Smoker	0.043	−0.12 to 0.21	0.61				
FHx of CAD	0.16	0.00061 to 0.32	0.05	FH of CAD	0.11	−0.054 to 0.28	0.18

Each variable was first entered into a univariable model, and those found to be significant at a level of P<0.20 were subsequently entered into a multivariable model. *PMR* plaque to myocardial signal intensity ratio, *LDL* low density lipoprotein, *HDL* high density lipoprotein, *FHx* family; *CAD* coronary artery disease

Data are expressed as B-coefficient with 95% confidence intervals (CI) with P values.

**Fig. 6:**
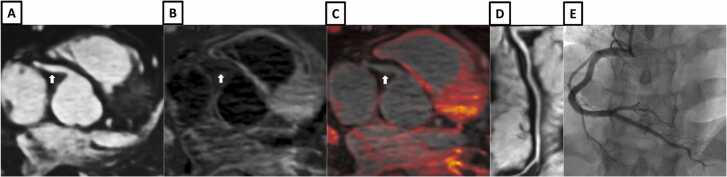
A patient with a normal RCA (white arrows). **A-**Bright-blood CMRA from iT2Prep-BOOST. **B-**Co-registered, black-blood iT2Prep-BOOST image. **C-**Co-registered fusion image of iT2Prep-BOOST bright-blood and black-blood datasets. **D-**CMRA curved multiplanar reformat of the RCA. **E-**invasive X-ray coronary angiography. *CMRA c*oronary magnetic resonance angiography, *RCA* right coronary artery

**Fig. 7:**
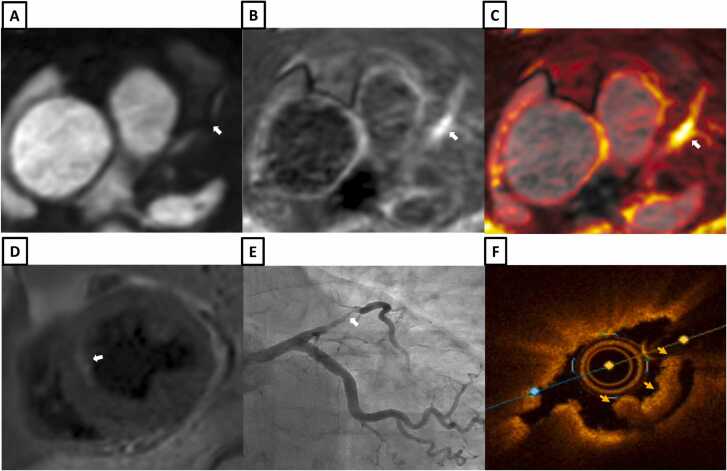
A patient with chest pain, peak high sensitivity troponin of 1993 ng/L and T-wave inversions in the anterior electrocardiogram leads. **A-**Bright-blood coronary angiography from iT2Prep-BOOST showing a stenosis in the proximal LAD (white arrow). **B-**Co-registered, black-blood iT2Prep-BOOST image showing increased signal corresponding to the culprit lesion (white arrow). **C-**Co-registered fusion image of iT2Prep-BOOST bright-blood and black-blood datasets showing increased signal from the culprit lesion (white arrow). **D-**Dark blood late gadolinium enhancement short axis image showing a partial thickness infarction affecting the basal antero-septal wall (white arrow). **E-**Invasive coronary angiography showing a severe stenosis in the proximal LAD (white arrow). **F-**Optical coherence tomography of the culprit LAD lesion demonstrating acute plaque rupture and intraluminal thrombus (yellow arrows). *LAD* left anterior descending artery

**Fig. 8:**
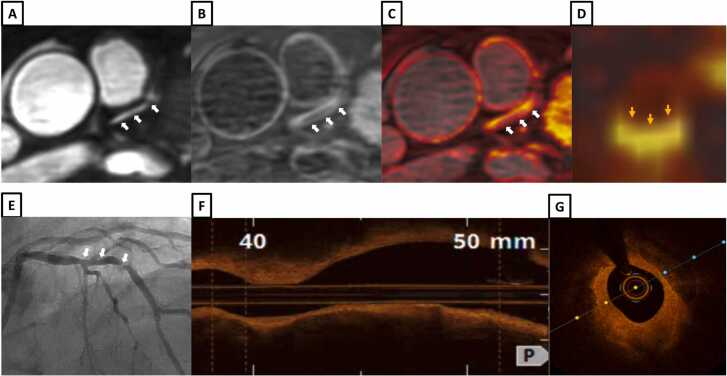
A patient with chest pain, peak high sensitivity troponin of 2173 ng/L and T-wave inversions in the anterolateral electrocardiogram leads. **A-**Bright-blood coronary angiography from iT2Prep-BOOST showing diffuse stenosis in the proximal to mid LAD (white arrows). **B-**Co-registered, black-blood iT2Prep-BOOST image showing increased signal corresponding to the stenotic lesions (white arrows). **C-**Co-registered fusion image of iT2Prep-BOOST bright-blood and black-blood datasets showing increased signal within the proximal to mid LAD (white arrows). **D-**Cross sectional view of the mid LAD lesion with the iT2Prep-BOOST fusion image (yellow arrows). **E-**Invasive coronary angiography showing diffuse stenosis in the proximal to mid LAD (white arrows). **F** and **G-**Optical coherence tomography of the mid LAD lesion demonstrating diffuse lipid rich plaques with fibroatheroma and calcification. *LAD* left anterior descending artery

**Fig. 9:**
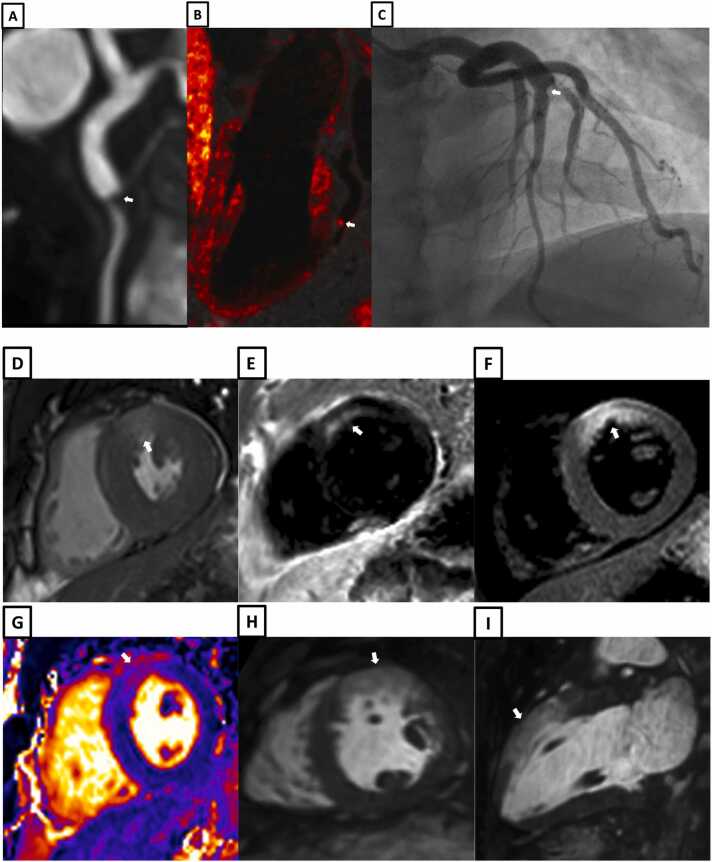
A 29-year-old patient with chest pain and peak high sensitivity troponin of 18269 ng/L. **A-**Multiplanar reformat of bright-blood coronary angiography from iT2Prep-BOOST showing a focal stenotic lesion in the proximal first diagonal artery (white arrow). **B-**Co-registered fusion image of iT2Prep-BOOST showing increased signal within the proximal first diagonal artery (white arrow). **C-**Invasive coronary angiography showing a stenosis in the proximal first diagonal artery (white arrow). **D-** Short axis cine slice showing a signal distortion in the mid anterior wall (white arrow). **E-**Short-axis dark-blood late gadolinium enhancement showing a partial thickness infarction affecting the mid anterior wall (white arrow). **F** and **G-**Short axis T2 weighted short Tau Inversion Recovery image and T2 mapping showing inflammation/oedema in the mid LAD territory (white arrows). **H**- Mid-myocardial short-axis slice reconstruction from the 3D IR-T2prep heartbeat dataset, showing potential for simultaneous myocardial tissue characterization (oedema/inflammation) with iT2Prep-BOOST (white arrow). **I**- 2 chamber slice reconstruction from the 3D IR-T2prep heartbeat dataset, showing potential for simultaneous myocardial tissue characterization (oedema/inflammation) with iT2Prep-BOOST (white arrow)

**Fig. 10:**
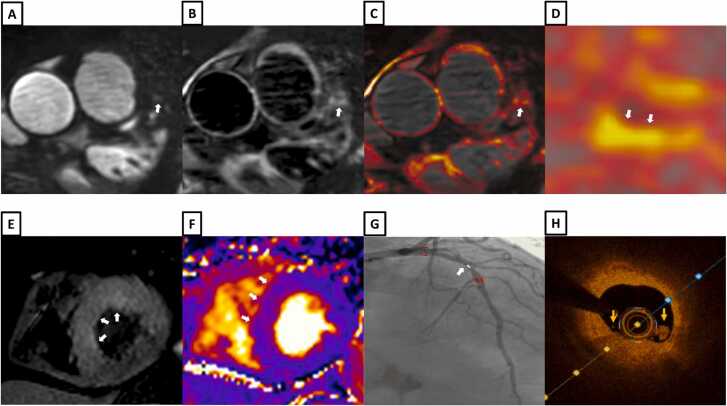
A patient with chest pain, peak high sensitivity troponin of 2309 ng/L and ST depression in the antero-lateral electrocardiogram leads. **A-**Bright-blood coronary angiography image from iT2Prep-BOOST showing a stenotic lesion in the mid LAD (white arrow). **B-**Co-registered, black-blood iT2Prep-BOOST image showing increased signal corresponding to the stenotic lesion (white arrow). **C** and **D-**Co-registered fusion image of iT2Prep-BOOST showing increased signal within the proximal to mid LAD (white arrow). **E** and **F-**Short-axis T2 weighted short Tau Inversion Recovery image and T2 mapping showing inflammation/oedema in the mid LAD territory (white arrows). **G-**Invasive coronary angiography showing a severe stenosis in the mid LAD (white arrow). **H-**Optical coherence tomography of the mid LAD demonstrating a lipid rich lesion with fibroatheroma and intraluminal thrombus (yellow arrows). *LAD* left anterior descending artery

**Fig. 11:**
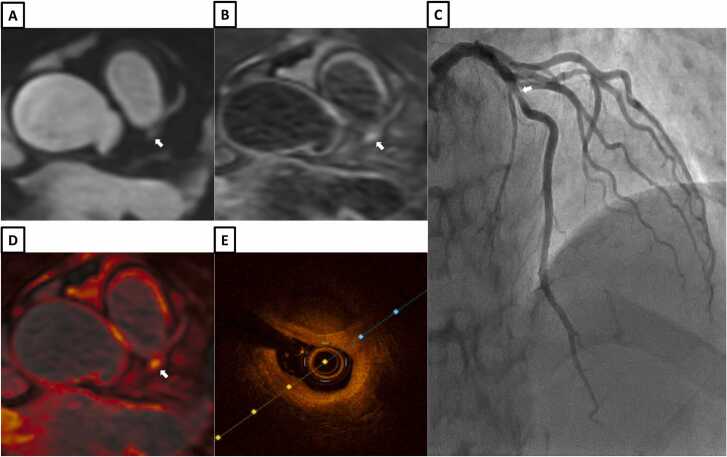
A patient with chest pain and peak high sensitivity troponin of 370 ng/L. **A-**Bright-blood coronary angiography image from iT2Prep-BOOST showing a stenotic lesion in the proximal LAD (white arrow). **B-**Co-registered, black-blood iT2Prep-BOOST image showing increased signal corresponding to the stenotic lesion (white arrow). **C-**Invasive coronary angiography showing a severe stenosis in the proximal LAD (white arrow). **D-**Co-registered fusion image of iT2Prep-BOOST showing increased signal corresponding to proximal LAD lesion (white arrow). **E-**Optical coherence tomography of the proximal LAD demonstrating a calcified, lipid rich lesion with fibroatheroma. *LAD* left anterior descending artery

**Fig. 12:**
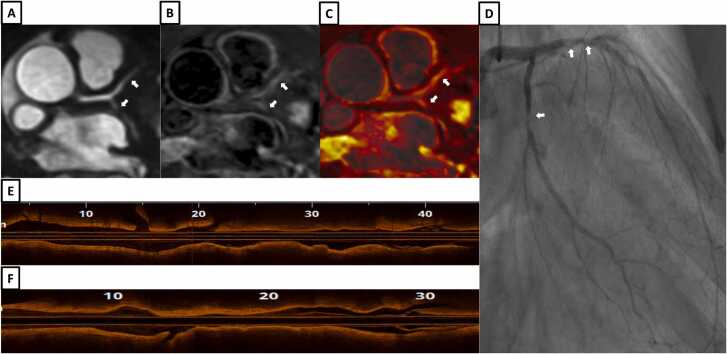
A patient with chest pain and peak high sensitivity troponin of 180 ng/L. **A-**Bright-blood coronary angiography image from iT2Prep-BOOST showing diffuse stenosis in the proximal to mid LAD and LCx (white arrows). **B-**Co-registered, black-blood iT2Prep-BOOST image showing increased signal corresponding to the stenotic lesions (white arrows). **C-**Co-registered fusion image of iT2Prep-BOOST showing increased signal corresponding to proximal to mid LAD and LCx lesions (white arrow). **D-**Invasive coronary angiography showing diffuse stenosis in the proximal to mid LAD and LCx (white arrows). Optical coherence tomography of the proximal to mid LAD **(E)** and LCx **(F)** demonstrating diffusely calcified, lipid rich lesion with fibroatheroma. *LAD* left anterior descending artery, *LCx* left circumflex artery

Example images from patients with iT2Prep-BOOST, invasive coronary angiography and intravascular imaging are shown in Fig. 6, Fig. 7, Fig. 8, Fig. 9, Fig. 10, Fig. 11, Fig. 12.

## 4. Discussion

In this study, we assessed for the first time the clinical value of iT2Prep-BOOST to simultaneously visualize coronary artery lumen and coronary plaque.

### 4.1. Coronary plaque characterisation at a segment level

We have demonstrated the feasibility of using iT2Prep-BOOST, based on PMR alone, to differentiate normal coronary segments from those with coronary atherosclerosis. This preliminary finding suggests that iT2Prep-BOOST has the potential to identify early atherosclerotic changes, which could play an important role in guiding therapeutic decisions. Additionally, our results indicate that iT2Prep-BOOST may be capable of distinguishing between culprit lesions, associated with acute coronary events, and non-culprit, bystander segments. This differentiation could be valuable for risk stratification, but further validation is needed in larger cohorts to confirm these initial findings.

Moreover, we have shown that it is possible to characterize coronary segments into groups based on their PMR values, allowing for the preliminary classification of plaques into specific categories, such as lipid-rich plaques, calcified plaques, and fibroatheromatic plaques, as well as more high-risk features like thin-cap fibroatheroma (TCFA), ruptured plaques, and thrombotic plaques. While these results are promising, they should be viewed as a feasibility demonstration, and further studies are required to fully validate the ability of iT2Prep-BOOST to reliably classify plaque types based on PMR.

In addition, we observed that PMR tends to increase with the degree of atherosclerotic burden, suggesting a potential correlation between PMR and disease severity. As the atherosclerotic burden increases, from mild stenosis to more advanced plaque, PMR values show a corresponding rise. However, larger studies with invasive and non-invasive imaging validation will be necessary to confirm this trend and establish PMR as a reliable marker for disease progression.

### 4.2. Patient level risk stratification

On a per-patient analysis, we observed an association between PMR and coronary artery risk factors, though HbA1c and family history of CAD were the only risk factors significantly associated with a higher mean PMR in multivariate analysis. While other risk factors did not show significant associations, this may be due to the sample size or the complex interplay of cardiovascular risk factors. Further research is needed to explore the relationship between PMR and other biochemical markers.

Other groups have demonstrated the potential of coronary CMR T1-weighted imaging as a monitoring biomarker for lipid-lowering therapy [12]. Based on these results, iT2Prep-BOOST could potentially serve as a non-invasive tool to monitor end-organ response to cardiovascular and anti-diabetic therapies. However, while our findings suggest this possibility, additional longitudinal studies are required to assess the reliability of iT2Prep-BOOST in this context and its ability to track treatment effects over time. Further validation in larger patient populations will be necessary to fully establish its role as a biomarker for therapeutic monitoring.

### 4.3. Novelty of iT2Prep-BOOST for vulnerable plaque imaging

The panacea of vulnerable plaque detection is the ability to image features of pathology that predispose to plaque rupture. We know from prospective multicenter studies using invasive intravascular imaging that non-obstructive vulnerable plaque features (e.g. lipid rich necrotic core and TCFA) are significantly predictive of future major adverse cardiovascular events [29], [30]. However, this method of vulnerable plaque imaging is expensive and comes with associated risks of invasive complications, whilst smaller distal segments are not readily imaged. Non-invasive alternatives including CCTA (coronary calcification, positive remodelling and low attenuation plaque assessment) [31], coronary 18F-sodium fluoride (18F-NaF) positron emission tomography (PET) (direct visualisation of coronary microcalcification activity) [32] and more recently a novel glycoprotein IIb/IIIa receptor antagonist-based radiotracer 18F-GP1-PET (direct coronary thrombus visualisation) [33] have all been shown to correlate with acute plaque-rupture events.

However, they are limited by the use of ionizing radiation, iodinated contrast agents and the need for radiotracers. Therefore, beyond the initial scan, they have a limited role for longitudinal follow up scans and monitoring of plaque progression or response to therapeutical intervention. There is therefore a need for an alternative non-invasive vulnerable plaque imaging modality which is free of ionizing radiation or need for contrast agents.

While iT2Prep-BOOST does not achieve the spatial resolution of invasive intravascular imaging modalities like IVUS or OCT, which can directly visualize specific plaque features, it compensates by utilizing the PMR to partially characterize plaque through differential signal intensity. This approach provides valuable insight into plaque composition, such as identifying fibroatheromatous or thrombotic plaques, even though the individual plaque components cannot be resolved at the microscopic level. The ability of PMR to serve as an indirect measure of plaque composition offers a promising alternative to invasive imaging techniques.

One of the key strengths of iT2Prep-BOOST is its non-invasive nature, which eliminates the risks associated with exogenous contrast agents and ionizing radiation, making it suitable for a wide range of patient populations and those requiring serial imaging, where cumulative radiation exposure is a concern.

Additionally, iT2Prep-BOOST can be integrated into comprehensive CMR protocols, enabling the simultaneous imaging of coronary arteries, myocardium, valves, great vessels, and pericardium within a single scanning session. This multifaceted capability allows clinicians to assess both plaque characteristics and cardiac function in a non-invasive and efficient manner, making it particularly useful for patients presenting with suspected ACS and in patients with stable symptoms. By combining plaque characterization with detailed myocardial evaluation, iT2Prep-BOOST has the potential to streamline the diagnostic pathway, reducing the need for multiple imaging sessions.

### 4.4. Novelty of iT2Prep-BOOST compared to CATCH

The iT2Prep-BOOST sequence builds on the foundations established by previous non-contrast CMR coronary plaque imaging sequences, including the CATCH sequence [9], [10], [11], which have been instrumental in advancing the field and literature of non-invasive coronary plaque imaging. iT2Prep-BOOST aims to further refine these capabilities.

The CATCH sequence utilizes an IR preparation pulse, optimized for detecting intraplaque hemorrhage and thrombus with high signal from these features and lower signal from the blood pool and vessel wall [10], [11]. While this approach effectively highlights high-risk plaque features, it may offer more limited visualization of other plaque components and the vessel wall.

In contrast, iT2Prep-BOOST combines IR and T2 preparation pulses to enhance plaque and vessel wall visualization, as well as coronary lumen contrast. Additionally, iT2Prep-BOOST uses advanced rigid and non-rigid respiratory motion correction with an image navigator and incorporates a denoising framework to improve image quality. In our study, we have also demonstrated the potential of iT2Prep-BOOST for simultaneous tissue characterization, specifically for identifying edema and inflammation, as illustrated in Fig. 10. However, it's important to note that this capability was not systematically validated within this study. Further research is necessary to confirm the accuracy and reliability of iT2Prep-BOOST in tissue characterization and to establish its clinical utility.

However, in the absence of a direct head-to-head comparison between CATCH and iT2Prep-BOOST, it is difficult to make definitive conclusions about the relative performance of each sequence. This limitation should be addressed in future studies to better evaluate the strengths and applications of each approach.

## 5. Limitations

This study has limitations. Due to unique design features of iT2Prep-BOOST (high signal intensity of myocardium in dark-blook images), smaller distal coronary segments and segments in very close proximity to the myocardium (e.g. myocardial bridging) may potentially be more difficult to analyze. Higher spatial resolution in combination with faster acquisition times are required to improve the image quality of iT2Prep-BOOST. Furthermore, whilst it was the aim to image every coronary segment with intravascular imaging, this was not logistically possible. Finally, this study focused on an ACS population, with a high prevalence of disease burden and severity. iT2Prep-BOOST should also be assessed in a cohort of patients with stable pathology.

### 5.1. Future directions

Further technical improvements have been made to the iT2Prep-BOOST framework. These include automatic trigger delay and iNAV position detection in order to simplify the planning process, minimize user input, and reduce errors. The 3D patch-based denoising technique has been implemented inline on the scanner for newer versions of the research sequence. Furthermore, deep learning neural networks could be employed to enable undersampled and/or super-resolution reconstruction, thereby significantly accelerating image acquisition and reconstruction. Recent studies have demonstrated the feasibility of obtaining sub-millimeter spatial resolution CMRA in less than 1 min or even within a single breathhold, approaching the acquisition time of CCTA, without compromising spatial resolution [34], [35], [36].

These features need to be assessed in a multicenter study of patients to establish the long-term prognostic value of iT2Prep-BOOST vulnerable plaque detection.

## 6. Conclusions

Coronary iT2Prep-BOOST has the potential to differentiate normal segments from non-culprit and culprit plaque segments in patients with suspected NSTEMI. Combined with conventional CMR, it could help facilitate earlier diagnosis and guide targeted intervention in patients presenting with suspected NSTEMI. The long-term prognostic value of PMR needs to be investigated in a large, prospective, multicenter study.

## Funding

The authors acknowledge financial support from: (1) King’s BHF Centre for Award Excellence RE/18/2/34213, BHF PG/18/59/33955, RG/20/1/34802 and FS/CRTF/20/24011 (2) EPSRC EP/V044087/1, EP/P001009/1, EP/P032311/1, EP/P007619, (3) Wellcome EPSRC Centre for Medical Engineering (NS/A000049/1), (4) Millennium Institute for Intelligent Healthcare Engineering ICN2021_004, FONDECYT 1210637 and 1210638, (5) IMPACT, Center of Interventional Medicine for Precision and Advanced Cellular Therapy, Santiago, Chile. ANID—Basal funding for Scientific and Technological Center of Excellence, IMPACT, #FB210024 (6) the Department of Health through the National Institute for Health Research (NIHR) comprehensive Biomedical Research Centre award, (7) NIHR Cardiovascular MedTech Co-operative and (8) the Technical University of Munich – Institute for Advanced Study. The views expressed are those of the authors and not necessarily those of the BHF, NHS, the NIHR or the Department of Health.

## Author contributions

**Reza Hajhosseiny:** Writing – review & editing, Writing – original draft, Visualization, Validation, Supervision, Software, Resources, Project administration, Methodology, Investigation, Funding acquisition, Formal analysis, Data curation, Conceptualization. **Adam Hartley:** Writing – review & editing, Validation, Resources, Methodology, Formal analysis, Data curation, Conceptualization. **Graham Cole:** Writing – review & editing, Validation, Supervision. **Camilla Munoz:** Writing – review & editing, Visualization, Supervision, Software, Resources, Methodology, Formal analysis, Conceptualization. **Amarjit Sethi:** Writing – review & editing, Validation, Supervision, Methodology. **Rasha Al-Lamee:** Writing – review & editing, Validation, Supervision. **Saud Khawaja:** Writing – review & editing, Validation, Supervision. **Sameer Zaman:** Writing – review & editing, Formal analysis. **James Howard:** Writing – review & editing, Validation, Supervision. **Deepa Gopalan:** Writing – review & editing, Validation, Supervision. **Ben Ariff:** Writing – review & editing, Validation, Formal analysis. **Raffi Kaprielian:** Writing – review & editing, Validation, Supervision. **Radhouene Neji:** Writing – review & editing, Software, Resources, Methodology, Conceptualization. **Karl P. Kunze:** Writing – review & editing, Visualization, Validation, Supervision, Software, Resources, Methodology, Investigation, Conceptualization. **Amit Kaura:** Writing – review & editing, Software, Methodology, Formal analysis. **Claudia Prieto:** Writing – review & editing, Visualization, Validation, Supervision, Software, Resources, Project administration, Methodology, Investigation, Formal analysis, Conceptualization. **Ramzi Khamis:** Writing – review & editing, Visualization, Validation, Supervision, Software, Resources, Project administration, Methodology, Investigation, Funding acquisition, Formal analysis, Data curation, Conceptualization. **René M. Botnar:** Writing – review & editing, Visualization, Validation, Supervision, Software, Resources, Project administration, Methodology, Investigation, Funding acquisition, Formal analysis, Conceptualization.

## Declaration of competing interests

The authors declare the following financial interests/personal relationships which may be considered as potential competing interests. Reza Hajhosseiny reports financial support was provided by British Heart Foundation. Reza Hajhosseiny reports financial support was provided by Engineering and Physical Sciences Research Council. Reza Hajhosseiny reports financial support was provided by National Institute for Health and Care Research. If there are other authors, they declare that they have no known competing financial interests or personal relationships that could have appeared to influence the work reported in this paper.

## Data Availability

The data underlying this article will be shared on reasonable request to the corresponding author.
