# Supplementary material for: Free-breathing, non-contrast, three-dimensional whole-heart coronary magnetic resonance imaging for the identification of culprit and vulnerable atherosclerotic plaque
Source: J Cardiovasc Magn Reson. 2025 Apr 22;27(1):101898. doi: 10.1016/j.jocmr.2025.101898 (PMC12135368; doi:10.1016/j.jocmr.2025.101898)
Supplement: Supplementary file 1 — Supplementary material [file mmc1.pdf]

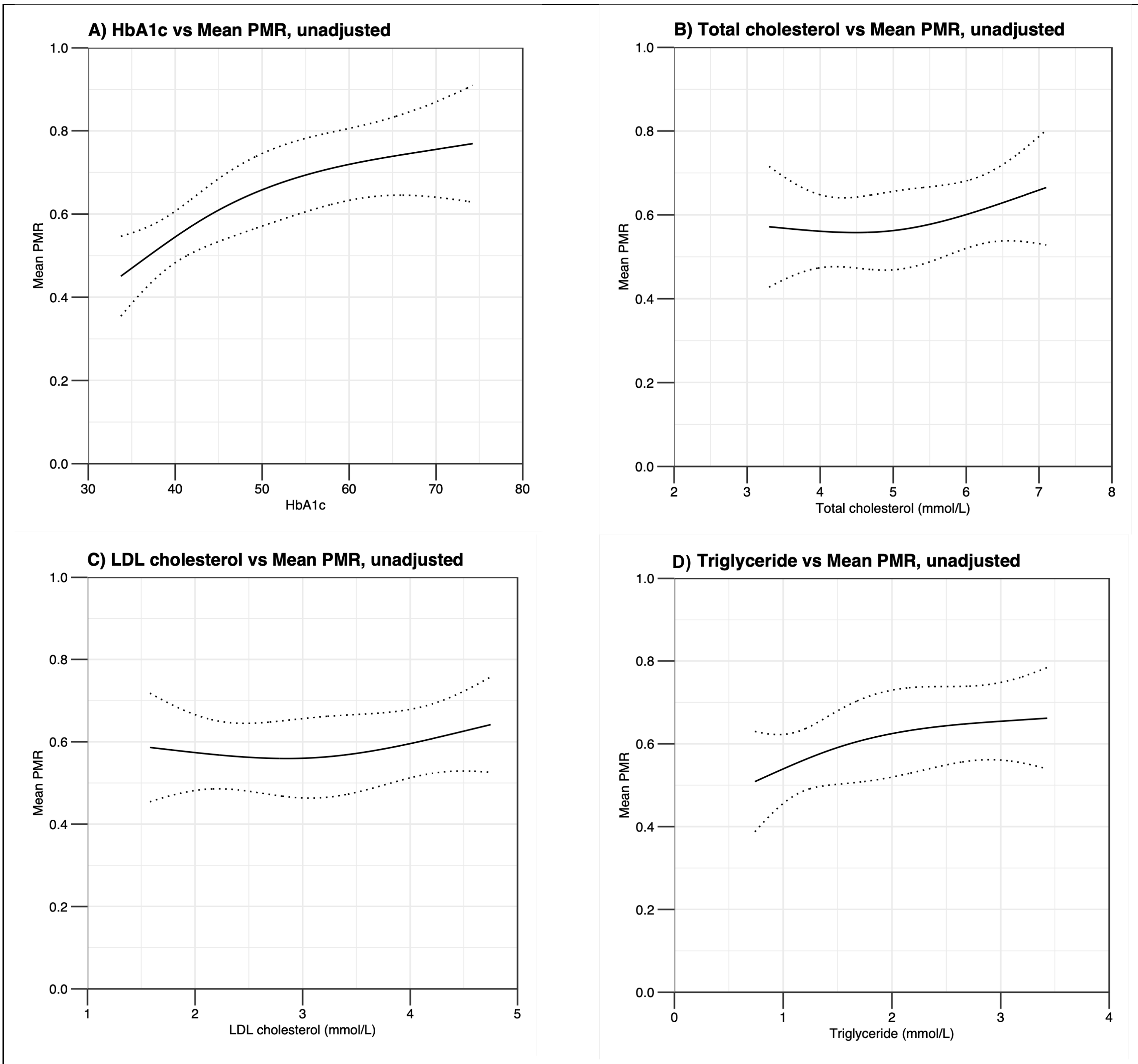

Supplementary Figure 1. Unadjusted spline curves of the association between biochemical cardiovascular risk factor and mean PMR

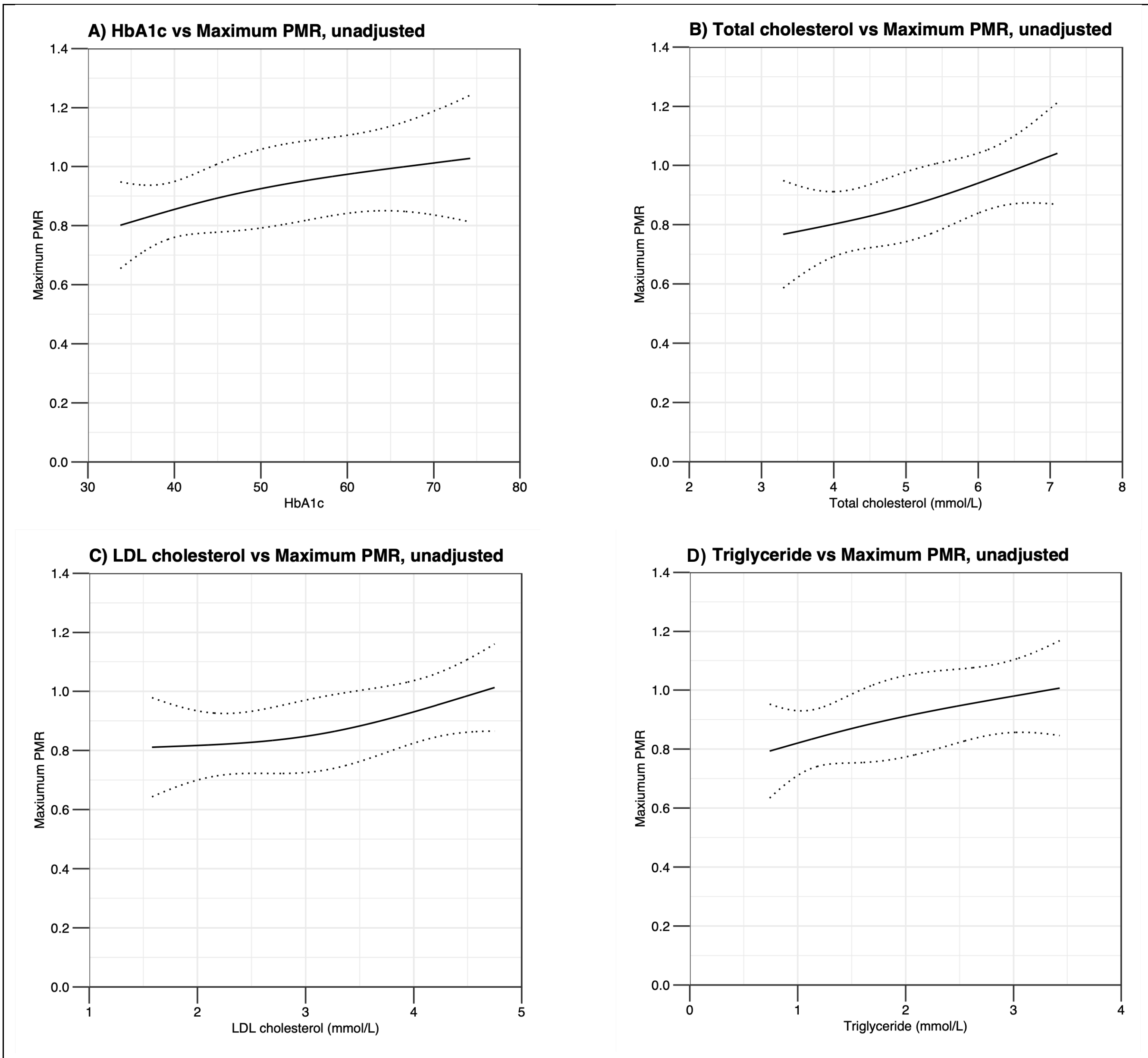

Supplementary Figure 2. Unadjusted spline curves of the association between biochemical cardiovascular risk factor and maximum PMR

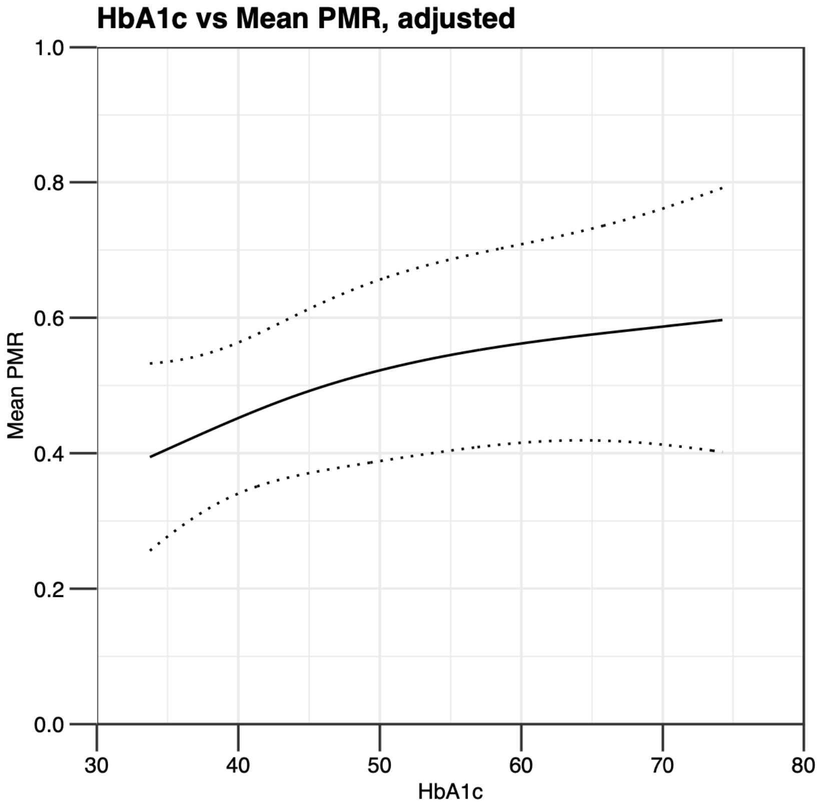

Supplementary Figure 3. Adjusted spline curve of the association between HbA1c and mean PMR

Adjusted for age, gender, total cholesterol, LDL cholesterol, triglyceride, hypertension, smoker and family history of coronary artery disease.

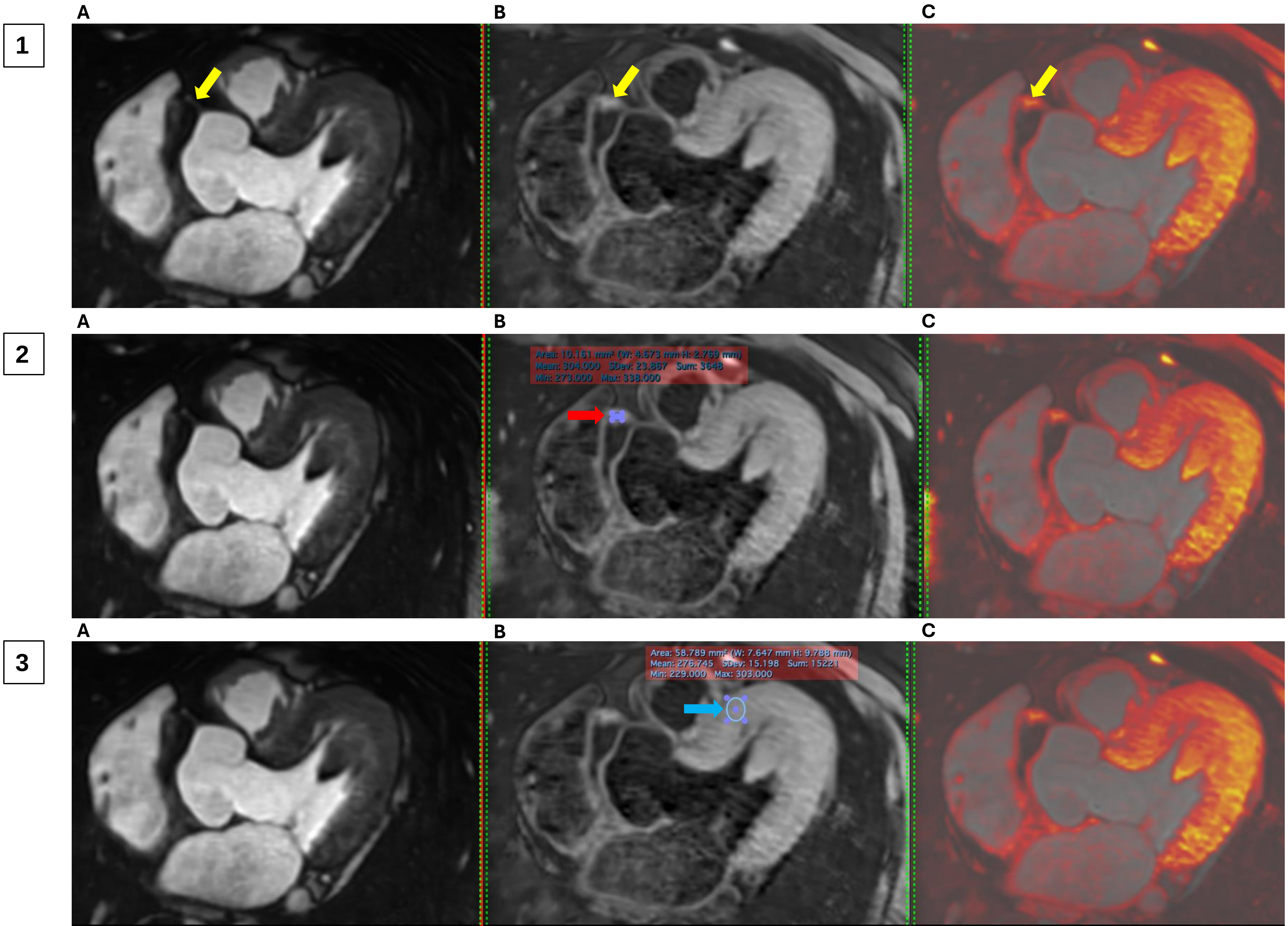
1
A
B
C
2
A
B
Area: 10.161 mm² (W: 4.673 mm H: 2.769 mm)
Mean: 304.000 SDev: 23.867 Sum: 3648
Min: 273.000 Max: 338.000
C
3
A
B
Area: 58.789 mm² (W: 7.647 mm H: 9.788 mm)
Mean: 276.745 SDev: 15.198 Sum: 15221
Min: 229.000 Max: 303.000
C

Supplementary figure 4: Example figure of how PMR was measured for each coronary segment. Row 1 – A: Bright blood anatomy dataset (odd heart beat) showing a severe stenosis in the proximal RCA (yellow arrow) for anatomical reference; B: Co-registered black-blood dataset (obtained by direct subtraction of even and odd heartbeats) showing a hyperintense signal corresponding to the same stenotic lesion in the proximal RCA (yellow arrow); C: Fusion feature of the Osirix workstation was used by overlaying the co-registered black-blood images onto the reference bright-blood CMRA images (even heart beat) for visualisation purposes only. Row 2 – A: The same bright blood anatomy dataset as row 1; B: Using the Osirix software region of interest (ROI) over the co-registered black-blood dataset hyperintense signal to obtain the plaque signal (mean plaque signal for this segment of 304.0); C: The same fusion dataset as row 1. Row 3 – A: The same bright blood anatomy dataset as row 1; B: Using the Osirix software region of interest (ROI) over the co-registered black-blood dataset to obtain the myocardial signal in the same plane (mean myocardial signal of 276.7, giving a PMR signal of 304.0/276.7=1.1); The same fusion dataset as row 1. The same steps were followed for all coronary segments, with the region of interest drawn around the plaque/vessel wall with the highest visualised signal on the co-registered black-blood and fusion datasets. PMR: Plaque to myocardial signal intensity ratio; RCA: Right coronary artery).
